# Diosgenin-Rich Yam Extract Enhances Cognitive Function: A Placebo-Controlled, Randomized, Double-Blind, Crossover Study of Healthy Adults

**DOI:** 10.3390/nu9101160

**Published:** 2017-10-24

**Authors:** Chihiro Tohda, Ximeng Yang, Mie Matsui, Yuna Inada, Emika Kadomoto, Shotaro Nakada, Hidetoshi Watari, Naotoshi Shibahara

**Affiliations:** 1 Division of Neuromedical Science, Department of Bioscience, Institute of Natural Medicine, University of Toyama, 2630 Sugitani, Toyama 930-0194, Japan; d1761202@ems.u-toyama.ac.jp (X.Y.); smily_flower000@hotmail.com (E.K.); hallotosay@yahoo.co.jp (S.N.); 2 Resilio Company Limited, Akasaka Minato-ku, Tokyo 107-0052, Japan; 3 Laboratory of Clinical Cognitive Neuroscience, Institute of Liberal Arts and Science, Kanazawa University, Kanazawa 920-1192, Japan; miematsui@staff.kanazawa-u.ac.jp (M.M.); yunainada@outlook.com (Y.I.); 4 Department of Japanese Oriental Medicine, Graduate School of Medicine and Pharmaceutical Sciences, University of Toyama, Toyama 930-0194, Japan; watari44@med.u-toyama.ac.jp; 5 Division of Kampo Diagnostics, Institute of Natural Medicine, University of Toyama, Toyama 930-0194, Japan; shiba1@inm.u-toyama.ac.jp

**Keywords:** diosgenin, *Dioscorea batatas*, cognitive function, healthy subject, Alzheimer’s disease

## Abstract

Diosgenin, a yam-derived compound, was found to facilitate the repair of axonal atrophy and synaptic degeneration and improve memory dysfunction in a transgenic mouse model of Alzheimer’s disease (AD). It was also found to enhance neuronal excitation and memory function even in normal mice. We hypothesized that diosgenin, either isolated or in an extract, may represent a new category of cognitive enhancers with essential activities that morphologically and functionally reinforce neuronal networks. This study aimed to investigate the effects of a diosgenin-rich yam extract on cognitive enhancement in healthy volunteers. For this placebo-controlled, randomized, double-blind, crossover study, 28 healthy volunteers (age: 20–81 years) were recruited from Toyama Prefecture, Japan, and was randomly assigned to receive either a yam extract or placebo. Preliminary functional animal experiments indicated that an oil solvent mediated the most efficient distribution of diosgenin into the blood and brain after oral administration, and was a critical factor in the cognitive benefits. Therefore, test samples (placebo and yam extract) were prepared with olive oil and formulated as soft capsules. The intake period was 12 weeks, and a 6-week washout period separated the two crossover intake periods. The Japanese version of the Repeatable Battery for the Assessment of Neuropsychological Status (RBANS) test was used for neurocognitive assessment, and the adverse effects were monitored through blood testing. Diosgenin-rich yam extract consumption for 12 weeks yielded significant increases in total RBANS score. Among the 12 individual standard cognitive subtests, diosgenin-rich yam extract use significantly improved the semantic fluency. No adverse effects were reported. The diosgenin-rich yam extract treatment appeared to safely enhance cognitive function in healthy adults.

## 1. Introduction

Alzheimer’s disease (AD) is a chronic, progressive, and untreatable neurodegenerative disorder characterized by the accumulation of amyloid β (Aβ) in the brain. Currently, clinically prescribed medicines for AD yield only slight improvements in symptoms or delays in disease progression [1]. Accordingly, several strategies aimed at reducing Aβ production have long been investigated in basic research and clinical trials centered around the radical development of therapeutic drugs for AD. However, these approaches have failed to improve cognitive function in phase 2 and 3 trials [2], even when supported by large pharmaceutical companies, and have been withdrawn. Possibly, these approaches may fail because Aβ accumulation in the brain starts approximately 30 years before the onset of symptoms, and has already plateaued once signs of AD become apparent [3].

We hypothesized that functional enhancement of the brain instead requires the reinforcement of neuronal networks, such as neurite regeneration and synapse formation. Our previous study of compounds with neurite regeneration activity found that diosgenin, a compound derived from several species of yam, repaired axonal atrophy and synaptic degeneration and improved memory dysfunction in a the 5XFAD transgenic mouse model of AD [4]. Very surprisingly, diosgenin treatment also enhanced object recognition memory in normal mice [5]. An in vivo electrophysiological study indicated that diosgenin treatment facilitated spike firing and cross-correlation in the medial prefrontal cortex and hippocampal CA1 in normal mice [5]. In addition, diosgenin-treated mice exhibited increases in axonal density and c-Fos expression in the medial prefrontal and perirhinal cortices, suggesting the enhancement of neuronal network activation. Mechanistically, diosgenin directly binds to and stimulates the membrane-associated rapid response steroid-binding receptor (1,25D_3_-MARRS) in neurons [4,5]. 1,25D_3_-MARRS is also expressed strongly in the human brain, particularly in cerebral cortical neurons, with moderate expression in the hippocampal neurons [6].

Findings from our preclinical studies suggest that diosgenin could strengthen cognitive function in healthy humans and possibly AD patients. Furthermore, recent subjects have focused on several compounds derived from natural medicinal components, such as *Gingko biloba* extract [7,8] and docosahexaenoic acid [9,10], as cognitive enhancers. However, only diosgenin has been found to promote neurite growth and reinforce neuronal networks. Diosgenin, therefore, may represent a new category of cognitive enhancer with the essential ability to support morphological and functional neuronal network reinforcement.

Certain yam species contain high levels of diosgenin, as well as diosgenin glucosides that are metabolized to diosgenin following oral intake [11]. In addition to our previous studies, a few animal studies reported that treatment with diosgenin [12] or a diosgenin-containing yam extract [13] ameliorated cognitive deficits in a mouse model of d-galactose-induced senescence. However, none of those studies evaluated the efficacy of diosgenin in humans. In this study, we aimed, for the first time, to investigate the effects of a diosgenin-rich yam extract on cognitive functions in healthy humans. A rodent experiment was conducted to determine the appropriate prescription for brain penetration of diosgenin after the oral administration of a diosgenin-rich yam extract.

## 2. Methods

### 2.1. Trial Design

**Figure 1:**
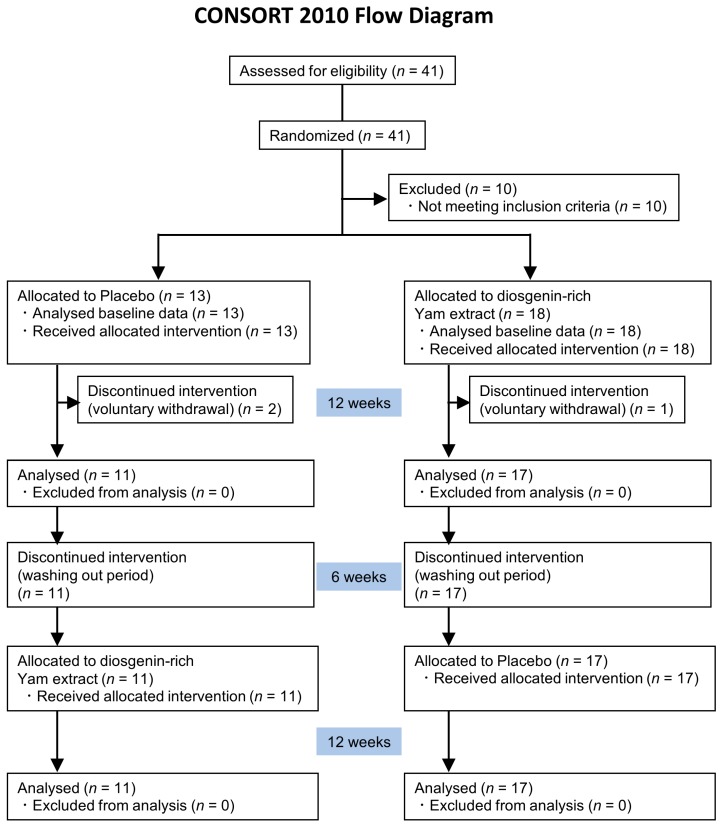
Study flow (CONSORT 2010 diagram). CONSORT, Consolidated Standards of Reporting Trials.

This placebo-controlled, randomized, double-blind, crossover study of healthy adults was conducted with the approval of the Ethics Committee of the University of Toyama. Each subject signed an informed consent form prior to study entry. The potential subjects (*n* = 41) were allocated into two groups. Thirty-one subjects who met the inclusion criteria were enrolled; after three discontinued the study for personal reasons, data from 28 subjects were finally analyzed. All of the subjects visited the University of Toyama four times for testing. Further details of the CONSORT flowchart of the study are shown in Figure 1.

### 2.2. Participants

The period of subject recruitment was from 12 December 2015 to 4 February 2016. The inclusion criteria for eligible subjects were as follows: (a) an age of ≥20 years; (b) facility with Japanese language; (c) residence in Toyama Prefecture, Japan; and, (d) good physical and mental health. The exclusion criteria were as follows: (a) diagnosis of AD or related disorders; (b) psychotic disorders; (c) cancer; (d) fewer than 12 years of education; (e) allergy to yam; and, (f) prescription for cognition-enhancing drugs or antipsychotics. Subjects were followed up from 1 March 2016 to 9 December 2016.

### 2.3. Intervention

The diosgenin-rich yam extract diopower 15 (Anti-Aging Pro Corporation, Tokyo, Japan) was used in this study. The extract was prepared from *Dioscorea batatas* (synonym *D. opposite*) on a large scale. Placebo capsules (two capsules/day = 672 mg olive oil (75% of ingredients), glycerol fatty acid ester, vitamin E derivative, white beeswax) and yam capsules)two capsules/day = 50 mg diopower 15 (5.6% of ingredients, 8 mg diosgenin), 672 mg olive oil (75% of ingredients), glycerol fatty acid ester, vitamin E derivative, white beeswax) were produced by the manufacturer (Shiratori Pharmaceutical, Narashino, Japan) under Good Manufacturing Practice controls and ISO22000 certification.

### 2.4. Outcomes and Assessments

All of the participants completed a basic sociodemographic and medical history questionnaire and reported any medications used at baseline. The Japanese version of the Repeatable Battery for the Assessment of Neuropsychological Status (RBANS) was administered as the primary neurocognitive outcome measure. The Japanese version of the Mini Mental State Examination (MMSE-J) was administered as a secondary outcome measure.

### 2.5. Neurocognitive Assessments

The RBANS, a representative, clinician-administered neuropsychological test for adults aged 20–89 years, was used to assess multiple cognitive function domains [14]. This test includes 12 standard cognitive subtests grouped into the following five domains: immediate memory (list learning and story memory), visuospatial/constructional (figure copy and line orientation), language (picture naming and semantic fluency), attention (digit span and digit symbol coding), and delayed memory (list recall, list recognition, story recall, and figure recall). The reliability and validity of the Japanese version of the RBANS have been well-established [15], and at least two forms have been prepared to avoid the effect of learning via test repetition. As noted above, the MMSE-J [16] was also applied. The Japanese Adult Reading Test (JART) was used to estimate the intelligence quotients (IQs) of the subjects as a background measure.

### 2.6. Safety Assessment

The safety assessment included the recording adverse events and conducting of biochemical blood tests to assess liver and renal function and blood sugar and lipid levels at each visit.

### 2.7. Randomization

The participants were randomly assigned to one of two groups. The first group consumed placebo capsules during the first round and yam capsules during the second round, whereas the second group consumed yam capsules during the first round and placebo capsules during the second round. Randomization was performed by a third-party company, CAC Croit Corporation (Tokyo, Japan), which secured the participant allocation list and performed key opening.

### 2.8. Animal Experiments: Animals and Materials

All of the animal experiments were performed in accordance with the Guidelines for the Care and Use of Laboratory Animals of the Sugitani Campus of the University of Toyama. All of the protocols were approved by the Committee for Animal Care and Use of the Sugitani Campus of the University of Toyama. The respective approval numbers for animal and gene recombination experiments are A2014-INM1 and G2013INM-1, respectively. Every effort was made to minimize the number of animals used. All of the mice were housed in a controlled environment (25 ± 2 °C, 50 ± 5% humidity, 12-h light/dark cycle starting at 7:00 a.m.) with free access to food and water.

Male (6 weeks old), male and female (9 weeks old), or female ddY mice (6 weeks old) purchased from Japan SLC (Shizuoka, Japan) were used in the experiments. Test compounds or vehicle solutions were administered intraperitoneally (i.p.) or orally (p.o.) once per day for 5, 4, or 7 days using an oral gavage. Transgenic mice (5XFAD) were obtained from the Jackson Laboratory (Bar Harbor, ME, USA) and maintained as double hemizygotes by crossing with B6/SJL F1 breeders. The effects of diosgenin were tested on both 5XFAD mice and non-transgenic wild-type littermates (male and female, 25–27 weeks old).

Diosgenin was purchased from Wako (Osaka, Japan). Diopower 15 was purchased from Anti-Aging Pro Corporation (Tokyo, Japan). All of the oils used in this study met the Japanese Pharmacopoeia quality standards.

### 2.9. Animal Experiments: Object Recognition Test

On the penultimate day of drug administration, the mice were individually habituated to a polyvinyl chloride open-field box (30 cm × 40 cm; height, 36.5 cm) for 10 min. On the last administration day, the mice performed a novel object recognition test, in which two identical objects (colored ceramic ornaments) were placed at a fixed distance within a square box (30 cm × 40 cm; height, 36.5 cm, 70–100 lux). A mouse was then placed at the center of the box, and the number of times the mouse contacted the two objects during a 10-min period was recorded (training session). The mice were again placed into the same box following a 48-h or 1-h interval after the training session, and one of the objects used during the training session was replaced with a novel object (another ceramic ornament with a different shape and color). The mice were then allowed to explore the box freely for 10 min, and the numbers of times the mice contacted each object were recorded (test session). A preference index, defined as the ratio of the number of times a mouse made contact with any object (training session) or with the novel object (test session) over the total number of times the mouse made contact with both objects, was used to measure the cognitive function for objects.

### 2.10. Animal Experiments: Brain Penetration of Diosgenin after the Oral Administration of a Diosgenin-Rich Extract

Diopower 15 (50 mg) was dissolved in 790 μL (672 mg) of olive oil or suspended in 790 μL of distilled water. Yam samples or vehicle solution (olive oil or saline) was orally administered using an oral gavage to female ddY mice (8 weeks old). The mice were euthanized at 3, 6, and 12 h after administration. Blood was collected and centrifuged at 10,000× *g* and 4 °C for 10 min to yield plasma. Plasma aliquots (200 μL) were extracted with methanol, dried, and resolubilized in methanol (100 μL). The cerebral cortex was also collected, homogenized, extracted with methanol, dried, sonicated, and resolubilized in methanol (100 µL).

To calculate the diosgenin concentration in the brain using liquid chromatography-mass spectrometry (LC-MS), a standard curve was generated by analyzing standard amounts of diosgenin solubilized in methanol. A Thermo Scientific Accela high-performance LC (HPLC) system, interfaced with an LTQ Orbitrap XL hybrid Fourier Transform Mass Spectrometer (Thermo Fisher Co., San Jose, CA, USA), was used to chemically profile diosgenin and the biosamples. The LC analysis was performed on a Capcell Pak C18 MGIII S-5 column (1.5 mm internal diameter × 150 mm, Shiseido, Tokyo, Japan) held at 40 °C with a flow rate of 200 μL/min. Ultrapure water (A) and ethanol (B) were used in the mobile phase, with the following linear elution gradient: 0–5 min, 65% B; 5–16 min, 95% B; 16–20 min, 55% B. The following electrospray interface (ESI) parameters were used: spray voltage, 4.5 kV; capillary voltage, 40.0 kV; tube lens, 150 V; capillary temperature, 330 °C; sheath gas flow rate, 50 units; aux gas flow rate, 10 units. We operated the mass spectrometer in the positive ESI mode with scanning from 50 to 2000 *m*/*z*, and calibrated the instrument using a polytyrosine solution before each experiment.

### 2.11. Statistical Analysis

The results are expressed as means with standard deviations (SD). Statistical comparisons were performed using GraphPad Prism 6 (GraphPad Software, La Jolla, CA, USA). All data were analyzed using two-tailed paired *t*-tests, and *p* values < 0.05 were considered significant.

## 3. Results

### 3.1. Baseline Characteristics of the Study Groups

The overall study population comprised of 28 healthy men and women. Figure 1 presents the CONSORT study flow diagram, subject distribution, and individual study protocols. Forty-one candidates were initially randomized into two groups. Following individual interviews, 10 subjects with fewer than 12 years of education were excluded. The remaining patients were allocated to a 12-week placebo period and 12-week diosgenin-rich yam extract period with a 6-week interval period, or a 12-week diosgenin-rich yam extract period and 12-week placebo period with a 6-week interval period. Three subjects (two and one in respective groups) withdrew from the study for personal, non-health-related reasons. Finally, 28 subjects completed all tests and were analyzed, and their characteristics are shown in Table 1. The analyses were done based on Intention-to-treat analysis.

### 3.2. Neuropsychological Functioning

**Figure 2:**
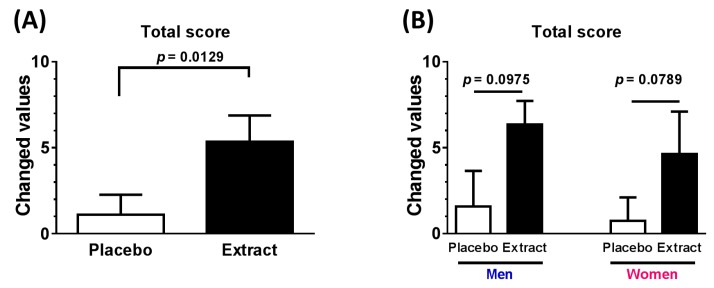

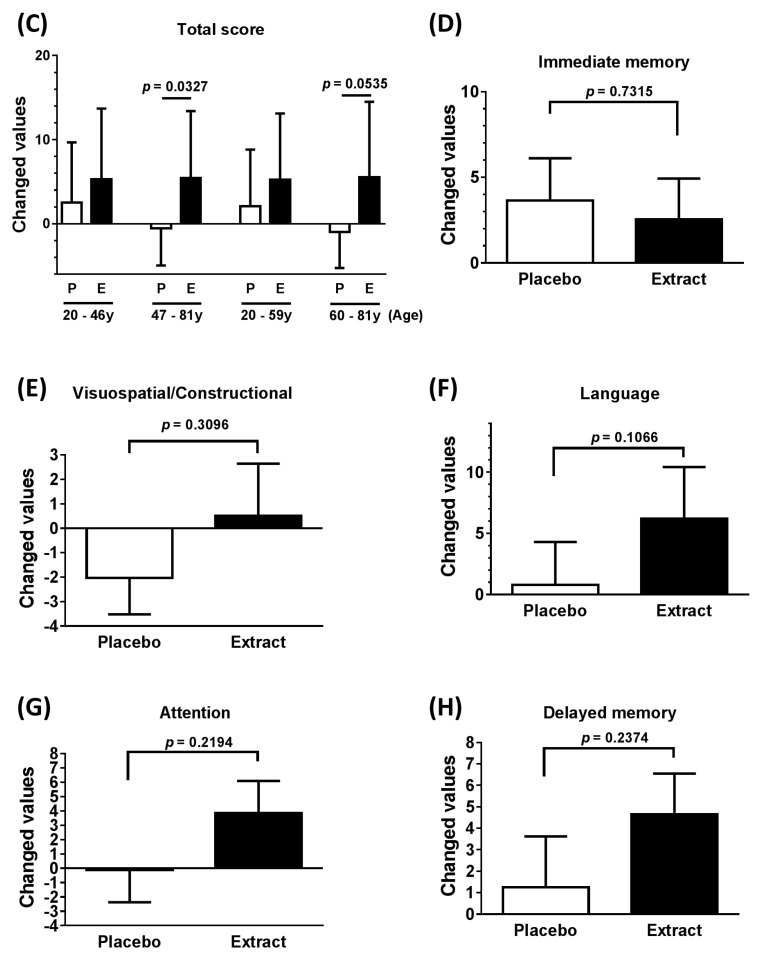
Effect of diosgenin-rich yam extract on cognitive function in RBANS test. Diosgenin-rich yam extract capsules or placebo capsules were taken for 12 weeks. RBANS total score in all participants (**A**), RBANS total score in each gender (**B**), RBANS total score in age ranges (**C**) P means placebo group and E means diosgenin-rich yam extract group. In (**D**–**H**), results of RBANS five domains were shown; Immediate memory (**D**), Visuospatial/Constructional (**E**), Language (**F**), Attention (**G**) and Delayed memory (**H**). *n* = 28 except (**B**,**C**). Men (*n* = 12) and women (*n* = 16) in (**B**). Age of 20–46 (*n* = 15), 47–81 (*n* = 13), 20–59 (*n* = 19) and 60–81 (*n* = 9) in (**C**).

The main analyses compared the RBANS total and five domain scores between the placebo and diosgenin-rich yam extract groups (Table 2 and Figure 2). A statistical evaluation of the changes in values from before to after the 12-week treatment period revealed that diosgenin-rich yam extract consumption led to a significant increase in the RBANS total score (Table 2 and Figure 2). This increase was independent of sex, but was age-dependent. Since the average of subjects’ age was 46.5 years, we divided 20–46 years group and 47–81 years group. Another grouping of 20–59 years and 60–81 years was also evaluated. Changed values in elder groups show significant effects of diosgenin-rich yam extract compared with placebo. Regarding the 12 individual standard cognitive subtests, diosgenin-rich yam extract was found to significantly improve semantic fluency (Table 3). The MMSE-J values were not affected by the placebo or extract (Table 2) because the baseline values were already near the maximum (29.39 ± 1.03).

### 3.3. Safety Measures

Thirty-one parameters were analyzed in blood to evaluate the incidence of side effects. Adverse effects were investigated following both placebo and diosgenin-rich yam extract intake. Table 4 lists the results of the safety evaluation. No adverse effects were shown in the diosgenin-rich yam extract intake. Interestingly, the triglyceride levels decreased with diosgenin-rich yam extract consumption.

### 3.4. Solubilization in Oil Facilitated Diosgenin Distribution in the Brain

**Figure 3:**
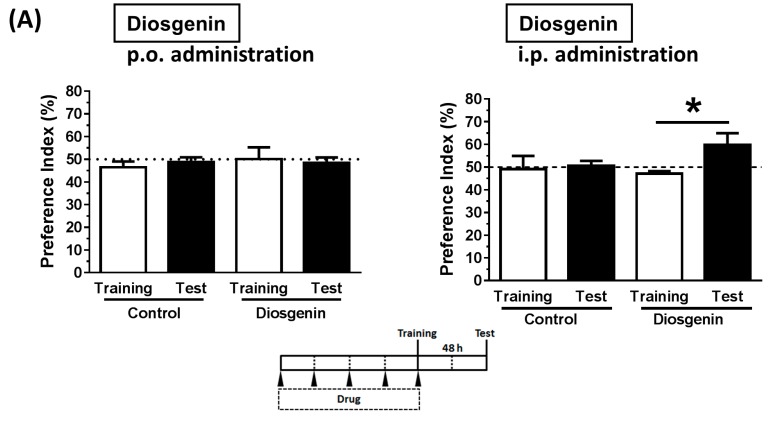

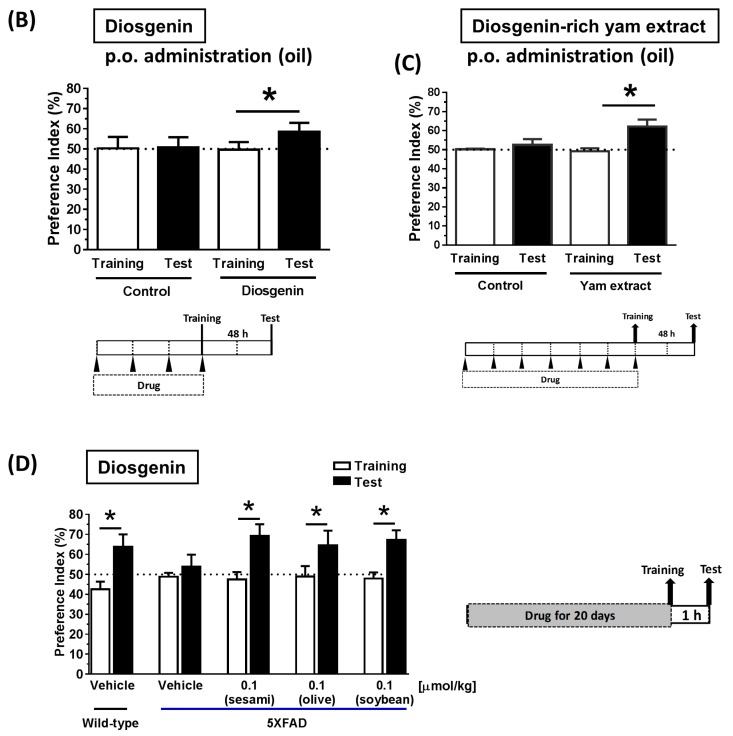
Object recognition memory test performances of treated mice. (**A**) Diosgenin was dissolved in a 10% ethanol + 5% glucose aqueous solution and administered to normal ddY mice (male, six weeks old) orally (p.o.) or intraperitoneally (i.p.) at a dosage of 10 μmol/kg/day (equivalent to 4.14 mg/kg/day). After consecutive treatment for five days, the novel object recognition memory test was performed with an interval of 48 h (*n* = 4); (**B**) Diosgenin was suspended in sesame oil and administered to normal ddY mice (male and female, 9 weeks old) p.o. at a dosage of 0.1 μmol/kg/day (equivalent to 0.0414 mg/kg/day). After consecutive treatment for four days, the novel object recognition memory test was performed with an interval of 48 h (*n* = 4); (**C**) Diosgenin-rich yam extract was suspended in soybean oil and administered to normal ddY mice (female, six weeks old). After consecutive treatment for seven days, the novel object recognition memory test was performed with an interval of 48 h (*n* = 3); (**D**) Diosgenin was suspended in sesame (*n* = 4), olive (*n* = 3), or soybean oil (*n* = 4) and administered to Alzheimer’s disease model mice (5XFAD transgenic; male and female, 25–27 weeks old) p.o. at a dosage of 0.1 μmol/kg/day (equivalent to 0.0414 mg/kg/day). As a vehicle solution, sesame oil was administered to wild-type (*n* = 5) and 5XFAD mice (*n* = 4). After consecutive treatment for 20 days, the novel object recognition memory test was performed with an interval of 1 h. * *p* < 0.05, two-tailed paired *t*-tests.

Diosgenin is a highly hydrophobic compound (cLogP; 5.7). To ensure complete dissolution in a water-based solution, diosgenin was first dissolved in ethanol to 10-fold of the final concentration, and this stock solution was subsequently diluted in a 5% glucose aqueous solution. The vehicle solution comprised of 10% ethanol in 5% glucose. Although we had previously investigated diosgenin treatments administered i.p. at a dosage of 10 μmol/kg/day (equal to 4.14 mg/kg/day) to mice [4,5], the p.o. administration of diosgenin had not been investigated. Figure 3A presents a comparison of the effects of diosgenin at a dosage of 10 μmol/kg/day (4.14 mg/kg/day) via the p.o. and i.p. routes on novel object recognition memory in normal mice. The consecutive administration of diosgenin i.p. for five days significantly enhanced object recognition memory, whereas p.o. treatment yielded no increase in memory ability.

Next, we tested sesame oil as a solvent for the lipophilic diosgenin. The consecutive p.o. administration of diosgenin for four days led to significantly enhanced object recognition memory in normal mice (Figure 3B). Diosgenin was dissolved directly in soybean oil by heating at 60 °C and stirring with a hand-held micro mixer. Surprisingly, this beneficial effect was achieved at a very low dose, 0.1 μmol/kg/day (0.0414 mg/kg/day). We also dissolved the diosgenin-rich yam extract in soybean oil for p.o. administration to normal mice for seven days at a set dosage (0.259 mg/kg/day, 16% diosgenin) to ensure an adjusted diosgenin dosage of 0.1 μmol/kg/day (0.0414 mg/kg/day). Again, this oil-dissolved yam extract significantly enhanced object recognition memory when administered p.o. (Figure 3C).

We next compared sesame, olive, and soybean oil solutions of diosgenin. These oil solutions were administered p.o. to 5XFAD mice at a dosage of 0.1 μmol/kg/day. Diosgenin was dissolved directly in sesame, olive, or soybean oil by heating at 60 °C and stirring with a hand-held micro mixer. After a 20-day administration period, the object recognition memory was evaluated (Figure 3D). Vehicle solution (sesame oil only)-treated 5XFAD mice exhibited no increase in the preference index. In contrast, treatment with diosgenin in sesame, olive, or soybean oil led to significant improvements in memory deficits in 5XFAD mice. The type of oil had no effect on memory improvement.

**Figure 4:**
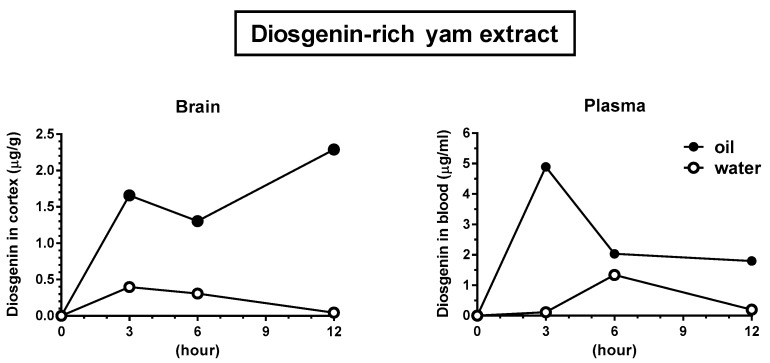
Distribution of diosgenin in the brain and plasma. Diosgenin-rich yam extract was suspended in olive oil or distilled water and administered to normal ddY mice (female, eight weeks old). Blood and brain tissue were collected before and 3, 6, and 12 h after extract administration, and the plasma and cerebral cortex samples were prepared. The diosgenin content of each sample was quantified using liquid chromatography-mass spectrometry. *n* = 1.

The functional evaluation of diosgenin (Figure 3A–D) indicated that the choice of solvent is a critical factor in mediating the cognitive benefits of oral administration. We further investigated the distribution of diosgenin in the blood and brain after the oral administration of oil and water-based solutions. As shown in Figure 4, oil solvents led to much higher and more persistent levels of diosgenin in the plasma and brain, compared with the water solvent. Neither vehicle solution (olive oil only or water only) yielded no diosgenin peaks in the plasma and cerebral cortex. We have subsequently registered our finding of the remarkable facilitation of diosgenin by oil suspension as a patent (patent number: P6165323).

## 4. Discussion

Various effects of diosgenin, including anti-cancer, anti-cardiovascular disease, anti-hyperlipidemia, anti-type 2 diabetes, and neuroprotective effects, have been investigated in both animal studies and in vitro studies [17]. However, studies have yet to report clinical evidence of the effects of diosgenin. In the present study, an orally administered diosgenin-rich yam extract was found to induce cognitive changes in healthy adults (20–81 years) according to the RBANS test outcomes (Table 2). Interestingly, elder subjects (more than 47 years) showed significant positive effects of diosgenin-rich yam extract in RBANS total score (Table 2 and Figure 2C). This study is the first to demonstrate the beneficial effects of a diosgenin-containing extract on cognitive functions in humans. The RBANS test was established and standardized carefully and provides a sensitive measure of changes in cognitive functions across a wide range of ages and populations. Among the RBANS subtests, we found that the diosgenin-rich yam extract especially enhanced semantic fluency within the language index (Table 3).

The low oral bioavailability of diosgenin has been well recognized. This low absorption has been attributed to the poor solubility of diosgenin in water, and a previous rat study therefore proposed the formation of a complex with cyclodextrin to increase solubility [18]. In our study, diosgenin dissolved in a 10% ethanol + 5% glucose solution had no effect on memory function in mice, despite successful solubilization (Figure 3A). In contrast, diosgenin dissolved in oil was efficiently distributed in the blood and brain (Figure 4), and exerted a memory enhancing effect both normal (Figure 3B,C) and AD model mice (Figure 3D). Hydrophobic compounds are generally absorbed by lymphatic vessels, and the use of an oil solvent might facilitate the lymphatic transfer of diosgenin. As a recent report identified lymphatic vessels in the brain [19], oil solutions of diosgenin might improve the utilization of this efficient delivery system.

In our study, the diosgenin-rich yam extract was administered at a dosage of 50 mg/subject/day, which corresponded to 8 mg of diosgenin/subject/day. Other commercially available dietary supplements based on a diosgenin-rich yam extract contain diosgenin at much higher dosages (20–50 mg/subject/day, >2.5-fold higher). Our results therefore indicate our ability to efficiently deliver low doses of diosgenin to the brain. Diosgenin itself appears to be a very safe compound, with a reported oral toxicity dose (LD_50_) of >8000 mg/kg in mice and rats (>480 g/human). Therefore, the supplementation with a diosgenin-rich yam extract should be both safe and effective.

In our previous mouse experiments, diosgenin treatment activated spike firing in neuronal circuits, as well as increased axonal densities in normal mice [5]. The present study findings suggest that a diosgenin-rich yam extract enhances cognitive function in healthy humans. Accordingly, we would like to conduct further human studies to obtain evidence of a relationship between increased neuronal excitation and axonal (white) matter, using modalities such as near-infrared spectroscopy (NIRS) or functional magnetic resonance combined with diffusion tensor imaging. We also expect that this diosgenin-rich yam extract might be effective for AD patients, given the improvements in memory observed in extract-treated AD model mice (Figure 3D). An upcoming clinical study will focus on the anti-AD effects of this extract.

## 5. Limitations

Several limitations of this investigation should be noted. We enrolled a well-educated sample of Asian adults, and therefore our results might not be generalizable to other populations. Furthermore, we did not assess the daily dietary intake and physical activity level, and are therefore unsure of the effects of these factors on the results of this study. The participants were advised not to modify their eating habits and activity patterns during the intervention. Finally, the study was also limited by the small sample size.

## 6. Conclusions

In conclusion, this placebo-controlled, randomized, double-blind, crossover study revealed that treatment with a diosgenin-rich yam extract enhanced cognitive function in healthy human adults without any adverse effects.

## Figures and Tables

**Table 1:** Sociodemographic and baseline characteristics of the sample.

	Values		
	*n*	Mean	SD
Subjects (Men/Women)	28 (12/16)		
Age (years)		46.50	18.67
Race	Asian		
Educated period (years)		16.29	2.37
Estimated intelligence quotients (IQ)		110.79	6.49
MMSE-J		29.39	1.03
Total cholesterol (mg/dL)		203.36	35.94

**Table 2:** Changes in Repeatable Battery for the Assessment of Neuropsychological Status (RBANS) scores between diosgenin-rich Yam extract intake and placebo intake.

Cognitive Domain	Mean of Difference	SD of Difference	95% CI	*p* Value
Total score (All)	4.250	8.949	0.974 to 7.526	0.0129 *
Total score (Men)	4.750	9.087	−1.023 to 10.520	0.0975
Total score (Women)	3.875	8.221	−5.056 to 8.256	0.0789
Total score (47–81 years)	6.000	8.963	0.5838 to 11.420	0.0327 *
Total score (60–81 years)	6.556	8.691	−0.125 to 13.240	0.0535 *
Immediate memory	−1.107	16.900	−7.659 to 5.445	0.7315
Visuospatial/Constructional	2.536	12.960	−2.489 to 7.560	0.3096
Language	5.429	17.210	−1.243 to 12.100	0.1066
Attention	3.964	16.690	−2.506 to 10.430	0.2194
Delayed memory	3.393	14.860	−2.369 to 9.154	0.2374
MMSE-J	−0.107	1.100	−0.5337 to 0.3194	0.6105

* *p* < 0.05, two-tailed paired *t*-tests.

**Table 3:** Changes in RBANS subtests scores between diosgenin-rich Yam extract intake and placebo intake.

Cognitive Domain	Subtest	Mean of Difference	SD of Difference	95% CI	*p* Value
Immediate memory	List learning	0.3214	3.1860	−9.1410 to 1.5570	0.5978
	Story memory	−0.6071	3.3260	−1.8970 to 0.6825	0.3426
Visuospatial/Constructional	Figure copy	0.1071	2.1140	−0.7126 to 0.9269	0.7906
	Line orientation	0.5000	2.4420	−0.4469 to 1.4470	0.2882
Language	Picture naming	0.1429	2.0850	−0.6658 to 0.9515	0.7198
	Semantic fluency	1.3210	3.1280	0.1087 to 2.5340	0.0338 *
Attention	Digit span	1.1070	4.2720	−0.5492 to 2.7640	0.1815
	Digit symbol coding	0.5357	3.3160	−0.7503 to 1.8220	0.4002
Delayed memory	List recall	0.0714	3.4950	−1.2840 to 1.4270	0.9147
	List recognition	0.0714	2.7070	−0.9783 to 1.1210	0.8900
	Story recall	0.3571	1.7890	−0.3366 to 1.0510	0.3002
	Figure recall	0.9643	3.2940	−0.3130 to 2.2420	0.1330

* *p* < 0.05, two-tailed paired *t*-tests.

**Table 4:** Changes in blood data between diosgenin-rich Yam extract intake and placebo intake.

	Changed Values				
	Placebo		Diosgenin-Rich Yam Extract		
	Mean	SD	Mean	SD	*p* Value
Total protein	−0.0393	0.3190	0.0464	0.3533	0.6102
Total cholesterol	−10.0000	22.9331	0.0357	25.1123	0.2765
HDL-cholesterol	−1.0357	13.3569	0.6786	8.9238	0.9651
Triglyceride	−13.8214	88.0980	−36.4286	72.4980	0.0674
Glucose (non-fasting)	−3.2143	29.0496	12.2500	28.1644	0.1105
AST (GOT)	1.4643	4.9026	−0.7500	4.2828	0.3281
ALT (GPT)	0.4643	5.4670	−0.1429	4.2922	0.9946
